# Cellulase Interacts with Lactic Acid Bacteria to Affect Fermentation Quality, Microbial Community, and Ruminal Degradability in Mixed Silage of Soybean Residue and Corn Stover

**DOI:** 10.3390/ani11020334

**Published:** 2021-01-28

**Authors:** Chao Zhao, Lihua Wang, Guangming Ma, Xin Jiang, Jinshan Yang, Jingyi Lv, Yonggen Zhang

**Affiliations:** College of Animal Science and Technology, Northeast Agricultural University, Harbin 150030, China; syszhaochao4474@163.com (C.Z.); Wanglihua4822@163.com (L.W.); 18846773453@163.com (G.M.); prozyg@sina.com (X.J.); yjs853396960@sina.com (J.Y.); 18846440487@163.com (J.L.)

**Keywords:** soybean residue, corn straw, lactic acid bacteria, cellulase, mixed silage, microbial community

## Abstract

**Simple Summary:**

The purpose of this experiment was to investigate the effects of lactic acid bacteria (LAB) and cellulase (CE) on the fermentation quality, bacterial community and ruminal degradability of soybean residue (SR) and corn stover (CS) mixed silage. The fermentation quality, rumen degradation rate and microbial diversity of mixed storage of SR and CS were effectively improved by adding lactic acid bacteria or cellulase, and the mixed addition of LAB and CE had a better effect, increased the abundance of beneficial lactic acid bacteria and reduced the abundance of harmful microorganisms, including Rahnella. In summary, in long-term mixed silage, the effect of mixed addition of lactic acid bacteria and cellulase was better than that of single addition.

**Abstract:**

The objective of this experiment was to investigate the effect of lactic acid bacteria (LAB) and cellulase (CE) on the fermentation quality, rumen degradation rate and bacterial community of mixed silage of soybean residue (SR) and corn stover (CS). The experiment adopted a single-factor experimental design. Four treatment groups were set up: the control group (CON), lactic acid bacteria treatment group (LAB), cellulase treatment group (CE) and lactic acid bacteria + cellulase treatment group (LAB + CE). Among them, the amount of added LAB was 1 × 10^6^ CFU/g, and the amount of added CE was 100 U/g. After 56 days of mixed silage, samples were taken and analyzed, and the chemical composition, fermentation quality, rumen degradation rate and microbial diversity were determined. The results showed that the pH of each treatment group was significantly (*p* < 0.05) lower than that of CON, while the lactic acid and ammoniacal nitrogen contents of each treatment group were significantly higher than that of CON, with the highest contents in the LAB + CE group. The contents of DNFom (Ash-free NDF), ADFom (Ash-free ADF) and DM in the LAB + CE group were significantly lower than those in the CON group, while the content of crude protein (CP) was significantly higher than that in the CON group. The in situ effective degradation rates of DM (ISDMD), DNF (ISNDFD) and CP (ISCPD) were all significantly (*p* < 0.05) higher in each treatment group than in the control group. The results of principal component analysis showed that the bacterial composition of the LAB, CE and LAB + CE groups was significantly different from that of the CON group (*p* < 0.05). Bacterial genus level analysis showed that the content of lactic acid bacteria was significantly higher in the LAB + CE group than in the other treatment groups (*p* < 0.05), while the content of undesirable bacteria was significantly lower than in the other treatment groups. The results showed that the addition of Lactobacillus and/or cellulase in mixed silage of SR and CS could effectively improve the quality of mixed silage fermentation, rumen degradation rate and microbial diversity, with better results when Lactobacillus and cellulase were added together, which provides new ideas for better application of SR and CS in dairy production.

## 1. Introduction

In recent years, there has been unprecedented interest in the efficient use of agricultural byproducts, as their application can significantly reduce feed production costs and manage crop-induced environmental problems [1]. Soybean residue, a byproduct of soybean processing, is rich in protein (25.4–28.4%) and is produced in large quantities in Asian countries, especially in China. However, the high moisture content of soybean residue makes it difficult to preserve for long periods of time even with silage, making it difficult to use as animal feed [2]. Interestingly, blending high moisture byproducts with dry crop byproducts is an effective solution to the undesirable problems associated with high moisture silage. Therefore, it is necessary to find a resource-rich and inexpensive dry byproduct to not only promote the feed industry but also alleviate the competition between animals and humans for food.

Corn straw, commonly produced in China, is a very abundant agricultural byproduct with high DM content [3]. However, corn straw fibers are not easily digested by animals, which makes them difficult to use as animal feed, and they are discarded directly or burned in large quantities. This not only causes environmental pollution but also leads to the underutilization of feed resources [4,5]. Mixing high-moisture SR with dry CS silage reduces the moisture content and eliminates some of the undesirable fermentation characteristics of mixed silage, such as undesirable odor and low digestibility, and these characteristics can improve some of the undesirable responses of cattle to forage [6]. Thus, this approach not only reduces the undesirable disposal and environmental impacts of SR and CS but also helps to provide low-cost and high-quality animal feed for ruminant-fed farms. However, the adequacy of the bacterial population is an important issue that affects the quality of silage [7].

Lactic acid bacteria (LAB) anaerobically convert water-soluble carbohydrates (WSCs) into organic acids, thereby lowering the pH and inhibiting the activity of harmful microorganisms, which can be used for large-scale mixed silage of high-moisture byproducts and dry byproduct feeds to preserve and enhance the nutritional value of feeds [8]. However, different strains have different responses to different environments, pH values, and effects, and their fermentation by combining strains can improve the fermentation efficiency, quality, aerobic stability, and antimicrobial capacity of mixed silage [9,10]. The addition of a mixture of CE and LAB in feedstuffs can degrade cellulose to monosaccharides or oligosaccharides, thus enhancing LAB propagation to achieve higher fermentation quality [11]. Next-generation sequencing (NGS) has been used to analyze and quantify the silage microbiota [12], providing a relevant basis for further investigation of mixed silage fermentation. As such techniques continue to be investigated, we propose combining SR, CS, and mixed silage with LAB and CE and using NGS methods to further illustrate the effects of this addition on the quality of SR and CS mixed silage.

Currently, the mixing of SR with CS through the addition of LAB and CE to silage has rarely been reported. Therefore, we hypothesized that the addition of LAB and CE would improve the quality of mixed silage of SR and CS. To test this hypothesis, the effect of LAB and CE addition on the quality, rumen degradation, and microbial diversity of mixed silage of SR and CS was evaluated in this experiment.

## 2. Materials and Methods

### 2.1. Silage Preparation

Corn was planted on April 16 at the Acheng Experimental Base of Northeast Agricultural University (127°04′ E, 45°52′ N), and CS was harvested on 5 October 2019. SR was from a soybean factory in Harbin. LAB (*Lactobacillus plantarum*: *Lactobacillus Lactobacillus* 1:1, 1 × 10^6^ CFU/g) were provided by the Ruminant Nutrition Institute of Northeast Agricultural University. CE was purchased from a biological company (10,000 U/g). SR and CS were mixed in proportion (SR:CS = 78:22, *w/w*) in glass pots and adjusted to 65% moisture content on the basis of fresh weight. A two-factor experimental design was used in the present study. Four experimental groups were set up, namely, the control (CON), lactic acid bacteria treatment group (LAB), cellulase treatment group (CE) and lactic acid bacteria + cellulase treatment group (LAB + CE). For each treatment group. The amount of LAB added was 1 × 10^6^ cfu/g and the amount of cellulase added was 100 U/g. The additives were dissolved in 10 mL of distilled water and sprayed on 2 kg of mixed silage with a microsprayer, and the control group was sprayed with 10 mL of distilled water. After mixing, the mixture was transferred to 28 fermentation bags (40 cm × 50 cm) with a one-way breather valve, and the bags were vacuum sealed, sealed with a food vacuum sealer, and fermented at room temperature (28 °C ± 3 °C) for 8 weeks. Five replicate bags were prepared for each group, and the weight of each bag was recorded before storage. Three uniform samples were taken from each bag after completion of fermentation. The first portion was used for microbial readings and microbial diversity analysis. The second portion was used to check the quality of the mixed silage fermentation, and the third portion was dried (65 °C for 48 h) and then passed through a 1 mm sieve in a grinder for chemical composition and rumen degradation rate.

### 2.2. Sample Analysis

#### 2.2.1. Fermentation Quality Analysis

A 20 g of the sample was placed in 180 mL of distilled water and mixed thoroughly, followed by overnight at 4 °C. Thereafter, the mixture was filtered through four layers of gauze, and then was filtered with qualitative filter paper. The filtrate was immediately measured by a Sartorius PB-10 pH meter. The filtrate was centrifuged at 12,000× *g* at 4 °C for 10 min, and the supernatant was filtered through a 0.22 µm filter membrane. The concentrations of acetic acid (AA), propionic acid (PA) and butyric acid (BA) in the filtrate were analyzed by gas chromatography (Shimadzu GC-2010, Japan) while NH_3_-N was determined by a phenol-sodium hypochlorite colorimetric method [13].

#### 2.2.2. Chemical Composition Analysis

The removed samples were dried in a 65 °C oven for 48 h and then ground through a 1 mm sieve with a grinder. The dry matter content of the samples was determined, and the dry matter recovery (DMR) was calculated according to the method described in AOAC (1990) [14]. The CP content of the samples was determined using a Kjeldahl nitrogen analyzer (Foss, 2300 Automated Analyzer, FOSS Analytical AB, Hoganas, Sweden). According to the method described by Van Soest [15], the NDF, ADF and ADL (Acid Detergent Lignin) of the sample were measured using a fiber analyzer (ANKOM), Ash-free NDF (NDFom) was determined by placing the final glass fiber filter with NDF residue in a furnace set at 535 °C for 2 h. The difference between NDFom and ADFom was used to calculate the hemicellulose content, and the difference between ADFom and ADL was used to calculate the cellulose content. WSCs were analyzed by the 3,5-dinitrosalicylic acid colorimetric method [16].

#### 2.2.3. Microbiological Analysis

After fermentation, the sample was stored in-80 degree refrigerator, and then microbiological analysis of the mixed silage was performed by plate counting [17]. First, 10 g of the sample was added into 90 mL sterilized normal saline to prepare the extract.afterwards, using air shaker to shake it for 2 h in 220 r/min in the condition of 37 °C, and then the extract was obtained. Second, the extracts were diluted to 9 gradients (10^−1^ to 10^−9^), and three optimal dilution multiples were selected for bacterial community counting. Finally, 100 µL of the dilutions of different concentrations were evenly coated on MRS agar and Difco Manual Violet Red Bile Agar (VRB) and then placed in an anaerobic incubator at a constant temperature of 30 °C for 48 h. LAB were counted. Consistent with the above procedure, yeast and mycobacterial counts were performed after incubation (72 h at 28 °C) on Rose Bengal agar (RBA) and PDA agar, respectively.

#### 2.2.4. Measurement of Rumen Degradation Characteristics

With the approval of the Laboratory of Ruminant Nutrition, Northeast Agricultural University, mixed silage samples were determined at the Acheng Experimental Base, Northeast Agricultural University as described by Hassanat et al [18]. The composition and main nutritional components of the diet for experimental dairy cows are shown in Table 1. The rumen degradation rate of SR and CS mixed silage was determined using the nylon bag method. First, the samples were removed from a −20 °C freezer and ground to pass through a 1 mm sieve, and 7 g of sample (DM) was weighed in triplicate in a nylon bag (8 × 13 cm; 50 μm pore size). The bags were placed in the rumen peritoneal sac at 2, 4, 8, 16, 24, 36, 48, and 72 h. The 0-hour samples were loaded into nylon bags in 37 °C water for 5 min and then subjected to the same treatment as the other bags. At the end of each time point, the nylon bags were removed from the rumen and manually washed under cold tap water until the rinse tap water was clear and free of any odor. The washed bags were then dried in an air oven at 65 °C for 48 h. Using the determination of the disappearance rate of DM, CP, and NDF in the rumen, the Dhanoa (1988) equation was used to determine the trophic kinetic parameters [19]: y = a + b (1 − e^−ct^), where y is the rate of protein degradation at the time of incubation, a is the rapidly degradable fraction, b is the potentially degradable fraction, c is the rate of degradation of the components, and t is the time point of incubation. Effective degradation rate: ED = a + b [c/(c + Kp)]. a, b, c as above, Kp is the outflow rate, kp = 0.031/h.

### 2.3. Microbial Diversity Analysis

Bacterial community analysis of mixed silage was carried out at Lc-bio Technologies Co., Ltd. (Hangzhou, China) by high-throughput sequencing. DNA was extracted from the samples using an MN NucleoSpin 96 Soi DNA kit (Gene Company Limited, Beijing, China) in accordance with the kit’s instructions. The DNA obtained from each sample was subjected to two-step PCR amplification to construct a small-fragment sequencing library. For the first amplification step, the 16S rRNA gene of V3-V4 was amplified by extracting DNA as a template (primers: 314F, 5’-CCTACGGGNGGCWGCAG-3’, 805R, 5’- GACTACHVGGGTATCTAATCC-3’). PCR was carried out using a Veriti 96-well PCR instrument (9902, ABI) with a 10 µL system as follows: 50 ng of genomic DNA, 0.3 µL of Vn F (10 µmol/L), 0.3 µL of Vn R (10 µmol/L), 5 µL of KOD FX Neo Buffer, 2 µL of dNTP (2 mmol/L), 0.2 µL of KOD FX Neo, and 2.2 µL of ddH_2_O. The PCR conditions were as follows: 98 °C for 30 s, 98 °C for 10 s, 54 °C for 30 s, 72 °C for 45 s, and 72 °C for 10 min 35 cycles. The Solexa PCR products were recycled from a 2.0% agarose gel and purified using an OMEGA DNA purification column. The purified products were quantified using a Quant-iT PicoGreen dsDNA assay kit in accordance with the kit’s instructions. Thereafter, the amplicons were sequenced on an Illumina HiSeq 2500 sequencing platform using the paired-end sequencing method. The original sequences obtained were spliced using FLASH software (version 1.2.11) to obtain the original tag data. The raw tags obtained were filtered using Trimmomatic software (version 0.33) to obtain high-quality clean tag data. UCHIME software (version 8.1) was then used to identify and remove chimeric sequences to obtain effective tags. The tags were binned into operational taxonomic units (OTUs) using the clustering program USEARCH (version 10.0) based on a 97% sequence similarity level [20]. The OTUs obtained were eventually used for taxonomic assignment. The representative sequences for each OTU were compared with the Silva (Release128, http://www.arb-silva.de) database to obtain taxonomic classification at the phylum, class, order, family, and genus levels. The relative abundances of taxa in the mixed silage were determined using QIIME software to compare the bacterial community composition in the mixed silage with different additives [21]. QIIME can also be used to calculate the diversity of Beta [22]. Richness and diversity indices were determined using MOTHUR software (version 1.30) to compare the bacterial diversity among different additives.

### 2.4. Statistical Analyses

Before analysis, microbiological data were transformed by log_10_ on the basis of FM. Thereafter, all data were subjected to 2-way ANOVA with the fixed effects of LAB, cellulase and the interaction effect of LAB × cellulase, using the GLM procedure of SAS (version 9.0, SAS Institute Inc., Cary, NC, USA). Tukey’s test was used for significance, where *p* < 0.05 indicates a significant difference, and *p* < 0.01 indicates a highly significant difference.

## 3. Results

### 3.1. Chemical Characteristics of Soybean Residue and Corn Stover upon Mixed Silage

The chemical composition and microbial community composition of SR and CS before mixed silage are shown in Table 2. The DM contents of SR and CS were 16.61% and 92.74%, respectively. In addition, the pH of both was higher than 6. The contents of NDF and ADF in CS were higher than those in SR. In addition, the WSC content in SR was relatively low at 2.3%. 2.41 log_10_cfu/g FM of LAB and 2.57 log_10_cfu/g FM of yeast were detected in SR, and the coliform bacilli (CB) count was lower than the detected level (<2.00 log_10_cfu/g FM). No mold was detected. CB, yeast and mold in CS were 3.04 log_10_cfu/g FM, 5.59 log_10_cfu/g FM and 6.43 log_10_cfu/g FM, respectively. The level of LAB was lower than the detection level (<2.00 log_10_cfu/g FM). After SR and CS were mixed in the ratio of 78:22, the pH was 6.52. The contents of DM, CP, NDF, ADF, and WSC were 34.89%, 10.83%, 57.82%, 23.03%, and 5.15%, respectively. Moreover, LAB, CB, and mold were lower than the finished level of detection (<2.00 log_10_cfu/g FM), but Yeast content was detected as 3.56 log_10_cfu/g FM.

### 3.2. Analysis of Cellulase and Lactic Acid Bacteria, Chemical Composition and In Situ Effective Degradability of Soybean Residue and Corn Stover upon Mixed Silage

The chemical composition of the mixed silage of SR and CS after silage is shown in Table 3. The DM of the CE group and the LAB + CE group was significantly increased (*p* < 0.05) compared to the CON group, there was no significant difference compared to LAB group (*p* > 0.05), no difference was noted for the DM on the 2-way interaction of LAB × CE.when the CON group was compared to the LAB group. The CP content of the LAB group, CE group and LAB + CE group was increased by 17.41%, 12.02%, and 20.49%, respectively, compared to that of the CON group, and no difference was noted for the CP on the 2-way interaction of LAB × CE.The NDFom content in the LAB treatment group was not significantly different compared to the CON group(*p* > 0.05), and both the CE and LAB + CE groups showed a decreasing trend in DNFom content compared with the CON group, with decreases of 16.41% and 18.74%, respectively (*p* < 0.05). No difference was noted for the NDF on the 2-way interaction of LAB × CE. Interestingly, the same trend is observed in ADFom, with the ADFom content in the CE groups and LAB + CE groups decreasing by 16.92% and 20.77%, respectively, compared with the CON group (*p* < 0.05). Compared with CON, the content rose by 4.43% in the LAB group. No difference was noted for the ADF on the 2-way interaction of LAB × CE. The Cellulose content in the LAB, CE and LAB + CE groups was significantly different from that in the CON group (*p* < 0.05), with the highest content in the LAB group and no significant difference between the CE and LAB + CE groups. Hemicellulose was significantly different from CON except for the LAB + CE group and CE groups (*p* < 0.05), and the LAB were not significantly different from CON (*p* > 0.05). No difference was noted for the cellulose and hemicellulose, on the 2-way interaction of LAB × CE. There was no significant difference in WSC content between the treatment groups (*p* > 0.05), but it was slightly lower than that of the CON group.

The addition of LAB and CE played a different role in the in situ effective degradability of DM, CP and NDFom in mixed silage The results showed that, except for the significant difference in ISDMDD relative to the LAB + CE group (*p* < 0.05), the difference between the LAB group and CE group was not significant compared with CON, which indicated that the addition of LAB + CE was more beneficial than a single additive to promote silage. Regarding the effective in situ degradation rate of DM in the system, in ISNDFD, all three treatment groups differed significantly (*p* < 0.05) from CON, with the highest ISNDFD in the CE group. We found an increase in ISCPD in the LAB, CE, and LAB + CE groups, but the difference between the LAB and LAB + CE groups was significant (*p* < 0.05) compared to the CON group, and the difference between the CE group and CON was not significant (*p* > 0.05). In addition, No difference was noted for the ISDMD, ISNDFD, ISCPD on the 2-way interaction of LAB × CE.

### 3.3. The Analysis of Cellulase and Lactic Acid Bacteria, Fermentation Characteristics and Microbiological Analysis of Soybean Residue and Corn Stover upon Mixed Silage

The results of dry matter recovery, pH, NH_3_-N, VFA and microbial communities of feedstuffs during the mixing process are shown in Table 4. Compared with the CON group, the dry matter recovery was significantly higher in the LAB + CE group (*p* < 0.05), while there was no significant difference between the LAB or CE groups (*p* > 0.05). No difference was noted for the DMR on the 2-way interaction of LAB × CE. The rate of pH decrease in the mixed silage process is one of the key indicators of successful mixed silage fermentation. The pH of each treatment group was significantly lower than that of the CON group (*p* < 0.05), and the LAB + CE group reached the lowest pH, with no significant difference between the LAB group and the LAB + CE group (*p* > 0.05). No difference was noted for the PH on the 2-way interaction of LAB ×CE. This indicates that the addition of either LAB or CE is beneficial to promote the rapid reduction of pH of the silage system, and the LAB + CE group is more helpful to maintain a lower pH state at the later stage of silage, thus improving the fermentation quality of the silage. Compared with the CON group, the NH_3_-N concentration in both the LAB and LAB + CE groups decreased significantly (*p* < 0.05). Significant differences were noted for the A on the 2-way interaction of LAB × CE. The lactic acid content in each treatment group increased significantly (*p* < 0.05), with the highest concentration in the LAB + CE group The content of acetic acid was significantly higher in each treatment group than in the CON group (*p* < 0.05) and was highest in the LAB + CE group. The propionic acid content was decreased in both the LAB and CE groups compared to the CON group, and no propionic acid was detected in the LAB + CE group. No butyric acid was detected except for the CON group.

The results of microbial community changes in silage are shown in Table 4. The colony-forming units (CFU/g) of LAB in each treatment group after mixed storage were 7.34 log_10_cfu/g FM, 8.03 log_10_cfu/g FM, 7.99 log_10_cfu/g FM, and 8.76 log_10_cfu/g FM. Except for 3.22 log_10_cfu/g FM, all other groups were below the detection limit (<2.00 log_10_cfu/g FM). Yeast was not detected in the LAB + CE group and was below the detection limit (<2.00 log_10_cfu/g FM) in the LAB and CE groups. Mold was not detected in any of the three treatment groups but was below the level of detection (<2.00 log_10_cfu/g FM) in the CON group.

### 3.4. Analysis of Cellulase and Lactic Acid Bacteria in the Microbial Communities of Soybean Residue and Corn Stover upon Mixed Silage

**Figure 1:**
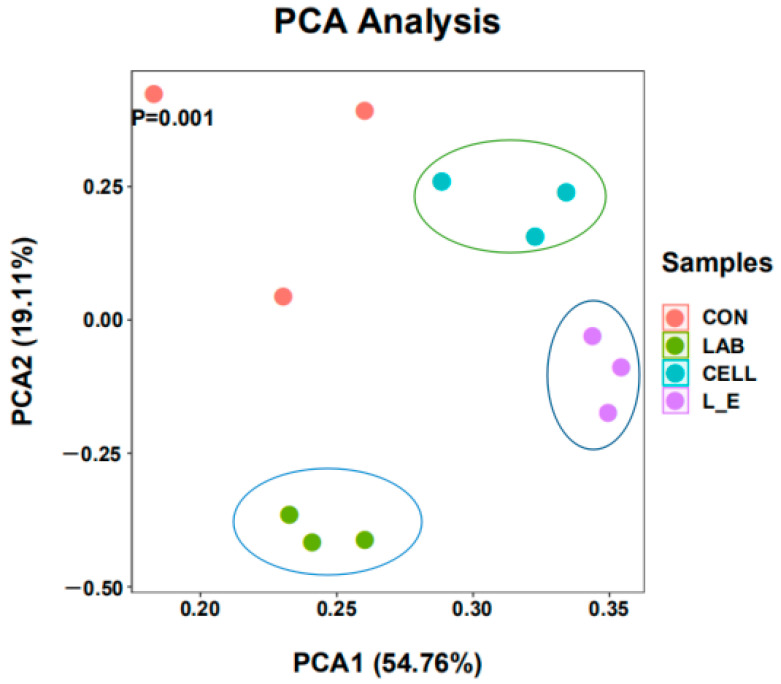
Principal component analysis of soybean residue and straw mixed silage bacterial community by cellulase and Lactobacillus. (Control = untreated feed; LAB = lactic acid bacteria; CE = cellulase; L_E = lactic acid bacteria +cellulase; 1, 2, 3, three replicates for each treatment).

The general sequence information for each treatment and the results of the bacterial diversity analysis are shown in Table 5. For all samples, the average coverage was >99%, which allowed further analysis of microbial community change. The Chao1 index was not significantly different among the treatment groups (*p* > 0.05). Additionally, the difference was not significant (*p* > 0.05) between the CE group and the LAB + CE group. The difference was that the CE group showed an increase in the Simpson index compared to the CON group (*p* < 0.05), and the LAB group and the LAB + CE group were not significantly different from the CON group (*p* > 0.05). Meanwhile, no difference was noted for the Shannon, Chao1, Simpson on the 2-way interaction of LAB × CE. To further investigate the changes in the microbial community environment, β-diversity analysis was performed, as shown in Figure 1, and principal component analysis clearly indicated the changes in the microbial community environment. The samples from the CON group were separated from the samples from the three treatment groups, indicating that the addition of Lactobacillus and cellulase had some effect on the microbial community of the mixed silage. Interestingly, the LAB, CE and LAB + CE groups did not show similar microbial communities.

**Figure 2:**
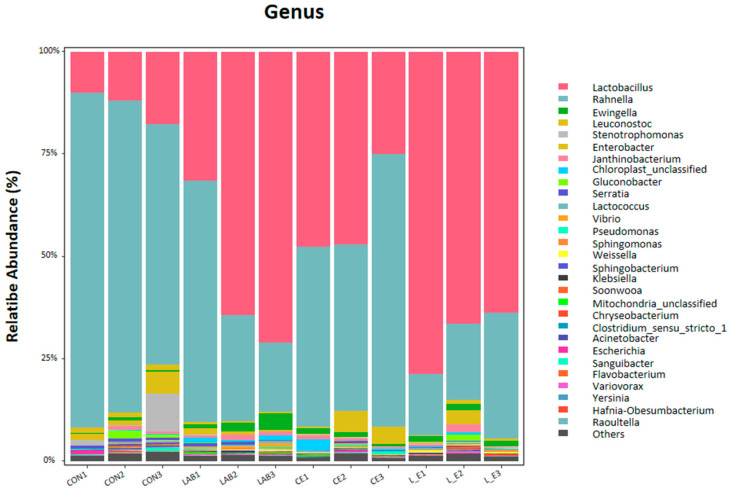
Cellulase and lactic acid bacteria in the bacterial community and the relative abundance of soybean residue and corn stover in mixed silage. (Control = untreated feed; LAB = lactic acid bacteria; CE = cellulase; L_E = lactic acid bacteria +cellulase; 1, 2, 3, three replicates for each treatment).

To further study the dynamic changes in the composition of the bacterial community at the genus level during the mixed silage process with lactic acid bacteria and cellulase, it can be seen in Figure 2 that the lactic acid bacteria at the genus level in the 56 d fermented silage sample mainly included Lactobacillus, Rahnella, Ewingella, Leuconostoc Stenotrophomonas, Enterobacter, and Janthinobacterium. The dominant lactic acid bacteria in each test were mainly Lactobacillus and Rahnella, and the relative abundance of Lactobacillus in the LAB + CE group was higher and significantly higher than that in the other groups, accounting for 63.29% of the total lactic acid bacteria community. The improvement in the quality of mixed silage between SR and CS may be due to the addition of additives that inhibit the activity of undesirable microorganisms and increase the number of beneficial microorganisms. Thus, the abundance of Rahnella was relatively high in the CON group (55.21%), but with the addition of LAB and CE, the number of Rahnella showed a decrease, accounting for only 19.65% in the LAB + CELL group. In addition, Ewingella and Leuconostoc were relatively low in the CON (1.01% and 0.41%), LAB (0.43% and 2.36%), CE (3.09% and 0.99%), and LAB + CE (0.57% and 1.24%) groups.

## 4. Discussion

### 4.1. The Chemical Characteristics and Microbial Population Prior to the Mixed Silage of Soybean Residue and Corn Stover

In general, moisture is one of the important factors affecting the fermentation quality of mixed silage, and different moisture contents may affect the growth and reproduction of microorganisms and thus the quality of the silage [23]. In this experiment, the moisture content of SR was similar to the previously reported range of 81.7–84.5% [24], which decreased to 65.0% when SR was mixed with CS. WSC is the key factor in silage fermentation when the WSC content ≥ 5% (DM) to ensure the quality of mixed silage fermentation [25]. In this experiment, although the WSC content of soybean residue was low, the addition of CS resulted in the lowest standard WSC content in mixed silage.

### 4.2. The Effects of Cellulase and Lactic Acid Bacteria on the Chemical Composition Analysis and In Situ Effective Degradability of Soybean Residue and Corn Stover in Mixed Silage

In this experiment, the DM content in both the LAB + CE groups was significantly higher than that in the CON group, which might be because LAB could produce more lactic acid in the mixing storage process, which led to a rapid decrease in pH and inhibited the growth of undesirable microorganisms, thus reducing DM loss [26]. The changes in WSCs as substrates for lactic acid fermentation in mixed storage directly reflect the utilization of WSCs by LAB and other microorganisms. In the conventional fermentation mode, the WSCs in the feed caused LAB to produce organic acids and significantly reduced the WSCs, which in turn rapidly created an anaerobic environment. This is consistent with the results of the Li test [27]. The WSC content in each treatment group was lower than that in the CON group, probably because LAB consumed a large amount of WSCs during the silage fermentation process, which promoted the rapid growth and reproduction of LAB and the production of organic acids, so the residual WSC content measured after 56 d of fermentation was also lower. NDFom and ADFom are the most effective indicators of fiber quality at this stage. ADFom and animal digestibility are negatively correlated. The lower the value of ADFom, the higher the digestibility of the feed and the higher the feeding value [28]. In this experiment, the NDFom and ADFom contents in the LAB + CE group were significantly lower than those in the CON group. On the one hand, this could be due to the CE addition promoting the degradation of fiber in SR and ML silage mixes; on the other hand, it could be due to the degradation of carbohydrates by LAB during silage fermentation to produce CO_2_, resulting in the reduction of fiber. This is consistent with the results of He’s test [29]. The results of Muck [30] showed that CE addition to LAB in mixed silage of high moisture amaranth and rice straw resulted in better chemical composition, bacterial community and aerobic stability than LAB alone.

The in situ effective degradation rate is an important parameter to measure the degree of nutrient fermentation in ruminants [31] because the CP in SR and CS is present in the cellular contents. Therefore, the degradation rate of protein is closely related to the proportion of the cell wall and cell content. The degradation rate of crude protein in the rumen can be determined more accurately by using the nylon bag method to put the sample to be tested in the rumen of dairy cows. In this experiment, the addition of LAB and CE was effective in increasing the ISCPD, probably because CE destroied the cell wall structure of the straw during fermentation, releasing nutrients and cellulose that promote the continuous growth of microorganisms. In the process, release more plant proteins and continue to synthesize new bacterial proteins that are more easily digested and absorbed by animals, which promotes digestibility and degradation [32]. The LAB + CE group significantly increased ISDMD and ISNDFD compared to the CON, LAB and CE groups. The reason may be that LAB degrades part of lignin and releases hemicellulose and cellulose, which in turn are broken down by rumen microorganisms into monosaccharides that are more easily absorbed by ruminants, accelerating the degradation of mixed silage in the rumen. In turn, this increases the effective in situ degradation rate of DM and DNF [33]. The above results indicate that LAB and CE addition can improve the nutritional value of SR and ML blends.

### 4.3. Effect of Cellulase and Lactic Acid Bacteria on the Fermentation Quality and Microbial Community of Soybean Residue and Corn Stover Mixed Silage

In the mixed silage fermentation process, the presence of undesirable microorganisms such as Clostridium perfringens can inhibit the growth of LAB, promote the production of butyric acid, and cause a deviation in the lactic acid fermentation route, which leads to protein degradation into NH_3_-N and consequently the loss of feed nutrients [34]. In this test, the concentration of NH3-N in the LAB + CE group was significantly lower than that in the CON group, probably because the addition of CE could degrade the fiber to WSCs, thus providing sufficient nutrients for LAB growth, promoting lactic acid production, and inhibiting the growth of undesirable microorganisms such as *Clostridium perfringens* in mixed silage. In addition, this hypothesis was confirmed by the fact that no butyric acid was detected in the LAB, CE and LAB + CE groups in this study (Table 4).

pH is an important indicator of the quality of silage fermentation [35]. Variation in pH depends on the silage DM, chemical composition, and silage cycle. Nishino et al. [36] found that the addition of LAB significantly reduced the pH of whole mixed ration silage, thereby improving its fermentation quality and aerobic stability. In this experiment, the pH of each treatment group was lower than 4.00, which may be because inoculation of LAB increased the initial number of lactic acid bacteria, thus promoting lactic acid production, which led to a decrease in pH and further inhibited the activity of spoilage organisms and protein hydrolysis [37]. The results of Zhang et al. showed that the addition of *Lactobacillus casei* lowered the pH of fescue silage, which is similar to the results of this study [38]. Organic acids are produced during silage fermentation, and these conditions can indicate the adequacy of the silage fermentation process. Liu et al. evaluated the effect of additives on the fermentation quality of TMR silage and found that the addition of *Lactobacillus casei* and cellulase to TMR silage resulted in a lactic acid yield of 3.8% (DM), which was similar to the lactic acid content (4.12%, DM) produced by the addition of LAB and CE blend silage in this experiment [39]. The acetic acid content was lower in each treatment group in this trial. This difference may be because the bacterial communities in the mixed silage came from different environments, their own characteristics, or the number of microorganisms on the raw materials [12].

In general, the need to apply additives to silage depends on the number and type of microorganisms attached to the silage [40]. For well-preserved mixed silage, the number of lactic acid bacteria in the mixed silage must be at least 1 × 10^5^ cfu/g [41]. However, the lactic acid bacteria in both soybean residue and straw were less than 1 × 10^5^ cfu/g and the undesirable microorganisms were high, which may lead to poor fermentation quality. Therefore, to ensure rapid silage fermentation and inhibit the growth of undesirable microorganisms, additives, such as LAB or CE, need to be added to enhance the ability of LAB to dominate microbial fermentation in the early stages of silage fermentation.

### 4.4. The Effects of Cellulase and Lactic Acid Bacteria on the Bacterial Community of Soybean Residue and Corn Stover upon Mixed Silage

Silage is composed of a community of microorganisms in a very complex environment [42], and bacteria play an important role in the whole silage process [43]. Lactobacillus is the main bacterial group that plays a major role in the silage process, and changes in the number and species of Lactobacillus are closely related to changes in the quality of silage materials [44]. In this study, the coverage value of each sample was approximately 0.99, indicating that most of the bacteria were detected. The Chao1 index of the CE group alone was higher, and the species diversity was richer for the same amount of sequencing; however, after the addition of LAB, the Chao1 index decreased, which may be because *Lactobacillus brucei* can improve the aerobic stability of silage materials during silage [45]. The LAB group exhibited lower diversity than the CON group with respect to the Shannon index. This may be due to the slow pH decline in high-moisture mixed silage, resulting in an inability to immediately suppress microbial activity, thus reducing bacterial diversity [9]. From the analysis in Figure 2, the LAB, CE, and LAB + CE groups all showed that LAB was the dominant microorganism at the end of the 56-day mixed silage, and the highest number of LAB microorganisms was found in the treatment group with mixed addition of LAB + CE. This indicates that mixed LAB and CE have a more stable microbial community than LAB or CE alone, thus contributing to the improvement of silage quality [46]. It has been widely reported that high levels of lactic acid bacteria (e.g., Lactobacillus spp.) are a key cause of rapid pH decline [35,47]. In this experiment, the abundance of Lactobacillus in the CON group was only 10.98%, and after the addition of LAB and CE, LAB in each treatment group dominated the bacterial community throughout the mixing process. Moreover, we found that the increase in Lactobacillus abundance was positively correlated with the increase in LA and AA, indicating that the increase in LA and AA was mainly caused by LAB. In this experiment, the genus-level bacterial communities in the three treatment groups evolved into 30 genera, including Lactobacillus, Rahnella, Ewingella, and Leuconostoc, in which the relative abundance of Lactobacillus and Enterobacteriaceae was higher, which is basically consistent with the reports of other authors [48]. Ostling and Lindgren (1991) successfully isolated Rahnella from wilted crops and showed that Rahnella is extremely regenerative. This may explain the high abundance of Rahnella in the control group [49]. On the other hand, the abundance of lactic acid bacteria increased significantly in the LAB + CE group after mixed silage, while the abundance of undesirable bacteria, such as Enterobacteriaceae, Salmonella and Campylobacter, decreased significantly in the LAB + CE group after the addition of both LAB and CE to the test.

## 5. Conclusions

The results of this experiment showed that the combined addition of LAB and CE increased the lactic acid and protein contents and decreased the NH_3_-N content and pH of the SR and CS blends, thereby improving their nutritional value. In addition, the application of LAB and CE to SR and CS blends increased the abundance of the desired Lactobacillus species and decreased the abundance of undesirable microorganisms. Thus, the combination of LAB and CE additions can significantly improve the quality of SR and CS silage blends, giving them the potential to be used in dairy production.

## Figures and Tables

**Table 1:** Composition and nutrient levels of the basal diet.

Items	Content
Ingredients	
Corn, % of DM	13.20
Wheat bran, % of DM	3.78
Molasses, % of DM	0.99
Soybean meal, % of DM	3.16
Distillers dried grains with solubles, % of DM	5.72
Cottonseed meal, % of DM	2.05
Corn gluten feed, % of DM	7.42
Corn germ meal, % of DM	4.51
Premix ^1^, % of DM	0.49
Corn silage, % of DM	15.70
Leymus chinensis, % of DM	42.98
Total	100.00
Nutrient levels ^2^	
Net Energy for Lactating/(MJ/kg)	5.44
CP	14.30
NDF	39.20
ADF	20.05
Ca	0.60
P	0.40

^1^ Premix: (% of DM) Ash 99.07%, Ca 14.27%, P 5.42%, Mg 4.96%, K 0.05%, Na 10.67%, Cl 2.98%, S 0.37%, Co 11 mg/kg DM, Cu 577 mg/kg DM, Fe 4858 mg/kg, I 51 mg/kg DM, Mn 1806 mg/kg DM, Se 13 mg/kg DM, Zn 1694 mg/kg DM, vitamin A115,240 IU/kg DM, vitamin D 46,100 IU/kg DM, vitamin E 576 IU/kg DM. ^2^ The net energy of lactation was calculated value, others were measured value.

**Table 2:** The chemical compositions and microbiological analysis of soybean residue and corn stover prior to mixed silage.

Items	Soybean Residue (SR)	Corn Stover (CS)	SR + CS
Chemical composition			
DM, % FW	16.61	92.74	34.89
CP, % DM	12.95	3.25	10.83
pH	6.68	6.15	6.52
EE, % DM	6.32	2.21	5.34
NDF, % DM	56.62	65.23	57.82
ADF, % DM	30.04	39.21	23.03
WSC, % DM	2.3	14.55	5.15
Microorganism			
LAB, log_10_ cfu/g FW	2.41	<2.00	<2.00
CB, log_10_ cfu/g FW	<2.00	3.04	<2.00
Yeast, log_10_ cfu/g FW	2.57	5.59	3.56
Mold, log_10_ cfu/g FW	ND	6.43	<2.00

NDF, neutral detergent fiber; ADF, acid detergent fiber; EE, ether extracts; CFU, colony forming units; CP, crude protein; DM, dry matter; FW, fresh weight; LAB, lactic acid bacteria; CB, coliform bacilli; ND, not detected; SD, standard deviation; WSC, water-soluble carbohydrates.

**Table 3:** The chemical compositions and in situ effective degradability of soybean residue and corn stover upon mixed silage on the condition of being added to lactobacillus and cellulases after 56 days.

Items	Treatment ^1^				SEM	*p*-Value ^3^		
	CON	LAB	CE	LAB + CE		L	E	L × E
Chemical Composition								
DM, % of FW	34.40 ^a^	33.64 ^a^	35.99 ^b^	35.22 ^b^	0.49	0.28	0.048	0.97
CP, % of DM	12.98 ^a^	15.24 ^b^	14.54 ^b^	15.64 ^b^	0.26	<0.01	0.03	0.15
NDFom, of DM	52.97 ^a^	54.73 ^a^	46.50 ^b^	44.61 ^b^	1.21	0.97	<0.01	0.32
ADFom, % of DM	31.92 ^a^	33.40 ^a^	27.30 ^b^	26.43 ^b^	0.71	0.77	<0.01	0.28
ADL, % of DM	7.59 ^a^	7.68 ^a^	6.14 ^b,c^	6.18 ^c^	0.04	0.09	<0.01	0.51
Cellulose, % of DM	24.33 ^b^	25.72 ^a^	21.16 ^c^	20.25 ^c^	0.15	0.02	<0.01	0.09
Hemicellulose, % of DM	21.05 ^b^	21.33 ^b^	19.2 ^a,b^	18.18 ^a^	0.32	0.21	0.17	0.35
WSC, % of DM	0.74	0.68	0.67	0.65	0.13	0.57	0.49	0.78
*In situ* effective degradability								
ISDMD, ^2^ % of DM	38.08 ^a^	40.11 ^a^	41.53 ^b^	42.19 ^b^	0.48	0.09	<0.01	0.35
ISNDFD, ^2^ % of NDF	16.53 ^a^	17.11 ^a^	18.97 ^b^	18.56 ^b^	0.44	0.90	0.01	0.45
ISCPD, ^2^ % of CP	24.72 ^a^	25.82 ^b^	24.79 ^a^	26.13 ^b^	0.27	0.01	0.63	0.76

NDFom, ash-free neutral detergent fiber; ADF, ash-free acid detergent fiber on an om basis; CP, crude protein; DM, dry matter; FW, fresh weight; WSC, water-soluble carbohydrates; SEM, standard error of the mean. ^a–c^ Means within a row with different superscripts differ from each other (*p* < 0.05). ^1^ Control = untreated feed; LAB = lactic acid bacteria (1 × 106 cfu/g); CE = cellulase (10,000 U/g); LAB + CELL = lactic acid bacteria (1 × 106 cfu/g) + cellulase (10,000 U/g). ^2^ ISDMD = in situ effective DM degradability; ISNDFD = in situ effective NDF degradability; ISCPD = in situ effective CP degradability. ^3^ Tukey’s test was used to detect differences between means at *p* < 0.05.

**Table 4:** The fermentation characteristics and microbiological analysis of soybean residue and corn stover upon mixed silage on the condition of being added to lactobacillus and cellulases after 56 days.

Items	Treatment ^1^				SEM	*p*-Value		
	CON	LAB	CE	LAB + CE		L	E	L × E
DMR, % of FW	96.51 ^a^	97.05 ^a^	97.65 ^ab^	97.98 ^b^	0.33	0.39	0.06	0.83
pH	3.98 ^a^	3.81 ^c^	3.92 ^b^	3.68 ^c^	0.01	<0.01	0.02	0.53
Ammonia-N, % of DM	6.92 ^a^	4.92 ^c^	5.73 ^b^	4.43 ^d^	0.01	<0.01	<0.01	<0.01
Lactic acid, % of DM	3.81 ^a^	4.98 ^c^	4.17 ^b^	5.45 ^d^	0.18	<0.01	0.02	0.83
Acetic acid, % of DM	0.75 ^a^	0.86 ^a^	0.81 ^a^	0.94 ^b^	0.03	0.19	0.30	0.77
Propionic acid, % of DM	0.17	0.11	0.16	ND	-	-	-	-
Butyric acid, % of DM	0.11	ND	ND	ND	-	-	-	-
Microorganism	-	-	-	-	-	-	-	-
LAB, log_10_ cfu/g FW	7.34 ^a^	8.03 ^b^	7.99 ^b^	8.76 ^c^	0.23	0.02	0.03	0.88
CB, log_10_ cfu/g FW	3.22	<2.00	<2.00	<2.00	-	-		
Yeast, log_10_ cfu/g FW	<2.00	ND	ND	ND	-	-		
Mold, log_10_ cfu/g FW	3.05	<2.00	<2.00	ND	-	-		

^a–c^ Means within a row with different superscripts differ from each other (*p* < 0.05). ^1^ Control = untreated feed; LAB = lactic acid bacteria (1 × 106 cfu/g); CE = cellulase (10,000 U/g); LAB + CELL = lactic acid bacteria (1 × 106 cfu/g) + cellulase (10,000 U/g).

**Table 5:** Diversity of alpha index values.

Sample ID	Treatment1 ^1^				SEM	*p*-Value		
	CON	LAB	CE	LAB + CE		L	E	L × E
Shannon Index ^2^	3.88 ^a^	3.37 ^b^	4.34 ^b^	3.94 ^a^	0.1	0.01	<0.01	0.71
Chao1 Index ^2^	213.79	215.75	239.35	232.25	14.18	0.33	0.90	0.83
Simpson Index ^2^	0.83 ^a^	0.81 ^a^	0.89 ^b^	0.86 ^ab^	0.02	0.21	<0.01	0.58
Good’s Coverage ^2^	0.99	0.99	0.99	0.99	-	-		

^1^ Control = untreated feed; LAB = lactic acid bacteria (1 × 10^6^ cfu/g); CE = cellulase (10,000 U/g); LAB + CELL = lactic acid bacteria (1 × 10^6^ cfu/g) + cellulase (10,000 U/g). ^2^ Chao was used to estimate the number of OTUs contained in the sample using the Chao algorithm; Simpson was used to estimate one of the microbial diversity indices in the sample; Shannon was used to estimate one of the microbial diversity indices in the sample. ^a,b^ Means within a row with different superscripts differ from each other (*p* < 0.05).

## Data Availability

The data presented in this study are available on request from the corresponding author.
